# Sex Disparity in Characteristics, Management, and In-Hospital Outcomes of Patients with ST-Segment Elevated Myocardial Infarction: Insights from Henan STEMI Registry

**DOI:** 10.1155/2022/2835485

**Published:** 2022-09-05

**Authors:** Shan Wang, You Zhang, Qianqian Cheng, Datun Qi, Xianpei Wang, Zhongyu Zhu, Muwei Li, Junhui Zhang, Dayi Hu, Chuanyu Gao, On behalf of Henan STEMI registry study group

**Affiliations:** ^1^Department of Cardiology, Central China Fuwai Hospital of Zhengzhou University, Henan Provincial People's Hospital Heart Center, Zhengzhou, China; ^2^Henan Institute of Cardiovascular Epidemiology, Zhengzhou, China; ^3^Henan Key Lab for Prevention and Control of Coronary Heart Disease, Central China Fuwai Hospital of Zhengzhou University, Zhengzhou, China; ^4^Institute of Cardiovascular Disease, Peking University People's Hospital, Beijing, China

## Abstract

**Background:**

Women hospitalized with ST-elevation myocardial infarction (STEMI) experience higher risk of early mortality than men. We aimed to investigate the potential impact of risk factors, clinical characteristics, and management among gender-related risk differences.

**Method:**

We analyzed 5063 STEMI patients prospectively enrolled from 66 hospitals during 2016–2018 and compared sex differences in mortality, death, or treatment withdrawal and main adverse cardiovascular and cerebrovascular events (MACCE) using the generalized linear mixed model, following sequential adjustment for covariates.

**Results:**

Women were older and had a higher prevalence of hypertension (53.3% vs. 41.1%, *P* < 0.001) and diabetes (24.5% vs. 15.2%, *P* < 0.001). Eligible women were less likely to receive reperfusion therapy (56.1% vs. 62.4%, *P* < 0.001); the onset to first medical contact (FMC) (255 vs. 190 minutes, *P* < 0.001), onset to fibrinolysis (218 vs. 185 minutes, *P* < 0.001), and onset to percutaneous coronary intervention (PCI) (307 vs. 243 minutes, *P* < 0.001) were significantly delayed in women. The incidence of in-hospital death (6.8% vs. 3.0%, *P* < 0.001), death or treatment withdrawal (14.5% vs. 5.6%, *P* < 0.001), and MACCE (18.5% vs. 9.4%, *P* < 0.001) were notably higher. The gender disparities persist in death (OR: 1.61, 95% CI: 1.12–2.33), death or treatment withdrawal (OR: 1.68, 95% CI: 1.26–2.24), and MACCE (OR: 1.37, 95% CI: 1.08–1.74) after adjustment for covariates. Among possible explanatory factors, age (−58.46%, −59.04%, −62.20%) and cardiovascular risk factors (−40.77%, −39.36%, −41.73%) accounted for most of the gender-associated risk differences.

**Conclusions:**

Women experienced worse in-hospital outcomes, and age and cardiovascular risk factors were major factors influencing sex-related differences. The sex disparity stressed the awareness and importance of quality improvement efforts against female patients in clinical practice.

## 1. Introduction

Coronary artery disease (CAD) is the leading cause of mortality in China and worldwide [1, 2]. ST-elevation myocardial infarction (STEMI) is one of the most severe CAD. Several studies showed that women had a greater risk of worse in-hospital outcome after STEMI [3–5], however, the effect of gender on mortality following STEMI have varied to some extent.

There is an ongoing debate on whether sex difference exists, or whether it is due to the presence of older age and comorbidities that cause the bad outcome of women after STEMI. Numerous studies have reported significant sex-based differences in short-term outcomes among STEMI patients [4, 6–10], however, some studies showed that gender was not an independent predictor of in-hospital mortality after adjustment for age and comorbidities [11–13]. Treatment time delay, older age, cardiovascular risk factors, comorbidities, and reperfusion therapy were suggested to be the major influence factors of in-hospital outcome [5, 14, 15]. The reasons for these inconsistent findings are numerous, such as the use of different populations with regard to age, differing times of patient recruitment, and adjustments for confounders.

In China, most patients are reluctant to die in a hospital, therefore, solely in-hospital death cannot represent the real severe outcome between sexes among patients with STEMI [16]. As such, it remains unclear whether sex differences in mortality persist in STEMI, whether the differences in in-hospital death or treatment withdrawal were consistent, and which factors may contribute to this gap in mortality and in-hospital death or treatment withdrawal.

The main purpose of this study was to investigate whether women with STEMI have worse in-hospital outcomes than men, and if so, to assess the role of age, cardiovascular risk factors, medical history, clinical characteristics, treatment delay, acute drugs, and reperfusion therapy in this excess of risk.

## 2. Methods

Data for this study were obtained from the Henan STEMI registry study (Unique identifier: NCT 02641262), which enrolled patients admitted with a definitive diagnosis of STEMI between September 2016 and August 2018, at 66 centers (50 secondary hospitals, 16 tertiary hospitals) from different geographic regions of central China (Henan province). The registry study has been registered at URL: https://www.clinicaltrials.gov, and the design has been described previously [17, 18].

### 2.1. Study Population

All STEMI patients enrolled in Henan STEMI registry were included. STEMI was defined in accordance with the universal definition of myocardial infarction (2012) [19], specifically as elevated biomarkers and new or presumed new ST-segment elevation >1 mm (0.1 mV) in two or more contiguous leads or new onset of left bundle branch block. Patients diagnosed as types 4a and type 5 STEMI and those transferred in with prior reperfusion (including intravenous or intracoronary fibrinolytic therapy and percutaneous coronary intervention (PCI)) from other hospitals were excluded. Finally, a total of 5063 STEMI patients (3829 males and 1234 females) were included in this analysis.

### 2.2. Data Collection

The baseline demographics, cardiovascular risk factors, medical history, clinical characteristics at admission, reperfusion therapy, and guideline-recommended acute drugs as well as in-hospital outcomes of consecutive STEMI patients were prospectively collected through a standardized online reporting platform with automatic checks for invalid values (Henan STEMI registry platform, Zhao Rui Corporation, Zhengzhou). We checked the consecutiveness of all cases, and a total of 53.8% of reported cases were audited for accuracy against medical records for on-site quality control.

### 2.3. Definition

Hypertension was defined as having a history of hypertension, or receiving antihypertensive therapy. Dyslipidemia was defined according to the guidelines for prevention and treatment of adult dyslipidemia in China as total cholesterol ≥ 5.2 mmol/L, LDL ≥ 3.4 mmol/L, or HDL ≤ 1.0 mmol/L [20]. Diabetes was defined as having a previous diagnosis of diabetes mellitus, or hemoglobin A1c level ≥6.5%. The current smoker was defined as smoking within the preceding year. A history of coronary heart disease was specified if patients had a clinical history of myocardial infarction or underwent PCI or coronary artery bypass grafting before the current hospitalization. The wall location of the MI was determined by electrocardiogram (ECG).

Patients eligible for reperfusion were defined as those with a primary diagnosis of STEMI, and admitted within 12 h after symptom onset. Contraindications for fibrinolysis were also recorded.

Treatment delays were considered from symptom onset to first medical contact (FMC, defined as the time of diagnostic ECG), FMC to fibrinolysis (from FMC to initiation of thrombolytic therapy), and FMC to PCI (from FMC to wire passage into the culprit artery).

### 2.4. In-Hospital Outcomes

The in-hospital outcomes include in-hospital death, death or treatment withdrawal, and main adverse cardiovascular and cerebrovascular events (MACCE). Considering that most Chinese patients are reluctant to die in the hospital, and treatment withdrawal is common, we take in-hospital death or treatment withdrawal as in-hospital outcomes, which can better reflect the prognosis of patients. MACCE includes all-cause death, treatment withdrawal, heart failure, nonfatal myocardial infarction, and ischemic stroke.

### 2.5. Statistical Analysis

We compared the baseline demographics, cardiovascular risk factors, medical history, clinical characteristics, reperfusion therapy, treatment delay, acute drugs, and in-hospital outcomes between women and men. For the descriptive purposes, categorical variables were presented as numbers and percentages, and were compared using the Chi-square or Fisher exact tests, whereas continuous variables were reported as mean and standard deviation (SD) and compared using the *t*-test, if normally distributed; or reported as median and interquartile range (IQR) and tested by Mann–Whitney *U* test, if not. All the analyses were performed considering differences in sex.

Generalized linear mixed models were fitted to estimate the odd ratios (OR) and corresponding 95% confidence intervals (CI) of in-hospital outcomes over different sex, which account for the clustering of patients within hospitals.

To understand the observed sex difference in study outcomes, unadjusted ORs were calculated with the base model that included only sex as the independent variable and accounted for the clustering of patients within hospitals. To understand the roles of each set of variables in explaining the sex difference in study outcomes, we added each set of variables one by one into the base model that accounts for the clustering of patients within hospitals to observe the change in the OR of sex, and further calculated the percent of the sex-associated risk difference accounted by each explaining variable [(the variable adjusted OR-unadjusted OR)/(unadjusted OR-1.0)*∗*100%]. Finally, we put all study variables into the final model to understand how much of the sex difference in study outcomes could be explained by all study variables in total.

The study variables included age, hospital grade (second hospital or tertiary hospital), socioeconomic status (marriage status and occupation), cardiovascular risk factors (hypertension, dyslipidemia, diabetes, and current smoker), medical history (coronary heart disease, heart failure, stroke, and chronic kidney diseases (CKD)), clinical characteristics (myocardial ischemia symptoms, heart rate, systolic blood pressure, Killip class, cardiac arrest, and anterior myocardial infarction at admission), time to present (onset-to-FMC), guideline-recommended acute drugs (aspirin, P2Y12 antagonists, and statin at emergency), and reperfusion therapy (successful reperfusion or not). We did all these analyses for in-hospital death, death or treatment withdrawal, and MACCE. Two-sided *P* values <0.05 were considered statistically significant. Statistical analyses were performed with SAS 9.4 (SAS Institute Inc., Cary, NC).

## 3. Results

Among 5063 patients with STEMI who were consecutively included in this study, 1234 (24.4%) were women, whereas 3829 (75.6%) were men. The median age of the study population was 63.1 years (IQR: 52.7–71.1), and 16.9% were older than 75 years.

### 3.1. Demographics and Cardiovascular Risk Factors

The baseline and clinical characteristics at the admission of the study population are summarized in Table 1. The median age of women was significantly older than men. Nearly one-third women were older than 75 years, whereas the proportion in men was 12.1%. Women were less likely admitted to a tertiary hospital; in the term of occupation, more than four-fifths female patients were farmers. Women had a higher prevalence of hypertension, diabetes, and heart failure, whereas men were more frequently to be current smokers and more likely to have a medical history of coronary heart disease. There was no significant difference in the medical history of dyslipidemia, stroke, or CKD.

### 3.2. Clinical Characteristics

Myocardial ischemia symptoms were usually atypical in female STEMI patients at admission, whereas 85.2% had typical symptoms in men. Furthermore, women had a higher percentage of anterior myocardial infarction and a lower proportion of Killip class I level. The proportion of heart rate ≥110 beats per minute and systolic blood pressure ≥140 mmHg was higher in female patients. On the side of the cardiac enzyme, the multiple of cardiac troponin I (cTnI) and creatine kinase-MB (CK-MB), which was calculated by the measured value divided by upper limit of normal value, were larger for female patients, while the creatinine and left ventricular ejection fraction (LVEF) were lower in male patients (Table 1).

### 3.3. Reperfusion Therapy

**Figure 1:**
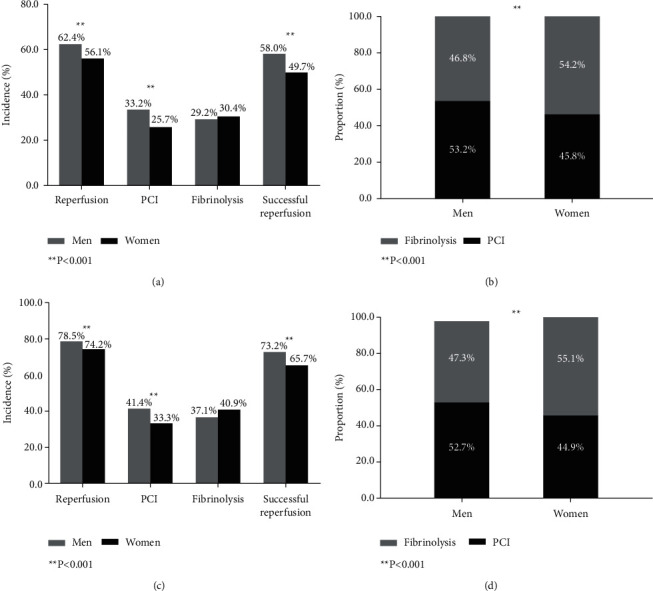
Use of reperfusion therapy based on sex in STEMI patients. In-hospital reperfusion strategy of all STEMI patients (240 patients with reperfusion contraindication were excluded) (a, b) and reperfusion eligible patients (admitted within 12 hours after symptom onset) (c, d). With regard to therapeutic strategies, 62.4% of male and 56.1% of female patients received reperfusion therapy (*P* < 0.001), PCI was performed in 25.7% of females versus 33.2% in males (*P* < 0.001), 49.7% of females versus 58.0% of males were successful reperfusion (*P* < 0.001) (a), and fibrinolysis was the major reperfusion therapy in female (b). Of the reperfusion eligible patients, 78.5% of male and 74.2% of female patients received reperfusion therapy (*P* < 0.001), PCI was performed in 33.3% of females versus 41.4% in males (*P* < 0.001), 65.7% of females versus 73.2% of male were successful reperfusion (*P* < 0.001) (c), and fibrinolysis was still the major strategy of reperfusion in female (d). STEMI, ST-elevation myocardial infarction; PCI, percutaneous coronary intervention.

In the analysis of reperfusion therapy, 240 patients were excluded for contraindications to thrombolysis. Among the 4823 STEMI patients without contraindications, 2940 (61.0%) have been submitted to reperfusion therapy. Women had a lower proportion of receiving reperfusion therapy compared with their counterparts, only a quarter of women were treated with PCI, and the proportion was significantly lower compared with men; and only about 49.7% of female patients underwent successfully reperfusion, which was also lower than man (Figure 1(a)). In respect to the reperfusion strategy, PCI seems to be the low-probability strategy in women (Figure 1(b)).

Among the 3682 eligible patients who presented within 12 hours, 807 (21.9%) were women; there were 599 (74.2%) female patients receiving reperfusion therapy and 530 (65.7%) were successful reperfusion, which was still lower than men—women still have lower successful reperfusion rate compared with their counterpart (Figure 1(c)). The main reperfusion mode for women was fibrinolytic therapy, which was contrary to men (Figure 1(d)). The above results were consistent with the overall analysis.

### 3.4. Treatment Delay and Acute Drugs

The median of onset-to-FMC time, onset-to-fibrinolysis time, FMC-to-fibrinolysis time, onset-to-PCI time, and FMC-to-PCI time is shown in Table 2. Onset-to-fibrinolysis time and FMC-to-fibrinolysis time were available in 1423 patients (341 in females and 1082 in males) who were given fibrinolytic therapy, and onset-to-PCI time and FMC-to- PCI time were available in 1517 patients (288 in females and 1229 in males) who received primary PCI. The median of onset-to-FMC time, onset-to-fibrinolysis time, FMC-to-fibrinolysis time, and onset-to-PCI time of females was significantly delayed than males. There was no difference in FMC-to-PCI time between the sexes. Time delays still exist in female patients.

In the terms of guideline-recommended acute drugs, women were less likely to receive aspirin, P2Y12 antagonist, dual-antiplatelet therapy (DAPT), and beta-blocker within 24 hours after being admitted. However, men were less likely to receive statin in an emergency.

### 3.5. In-Hospital Outcomes and Roles of Covariates in Explaining Sex Difference

**Figure 2:**
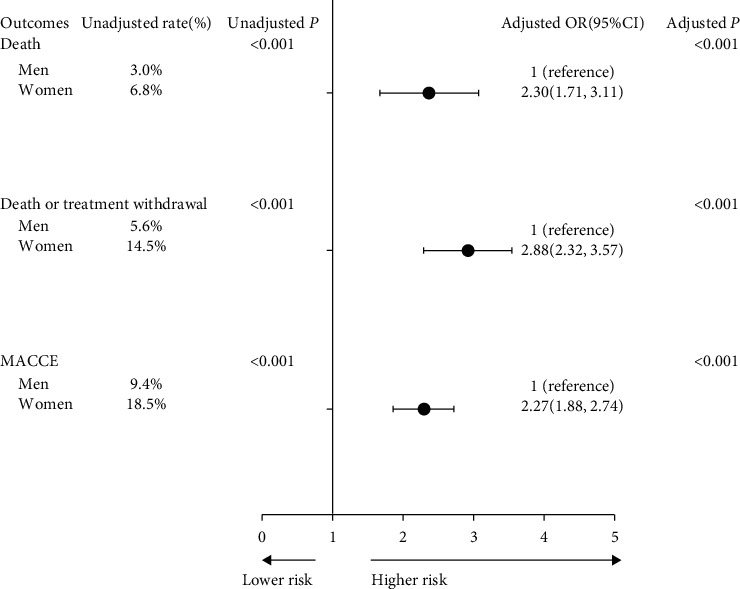
In-hospital outcomes based on sex in STEMI patients. The worse in-hospital outcomes were performed in female STEMI patients, and the differences between sexes were statistical significance in in-hospital death (6.8% in females vs. 3.0% in males, *P* < 0.001), death or treatment withdrawal (14.5% in females vs. 5.6% in males, *P* < 0.001), and MACCE (18.5% in females vs. 9.4% in males, *P* < 0.001). Adjustment for clustering of patients within hospitals in the generalized linear mixed model, and the sex differences were significant between men and women with respect to death, treatment withdrawal, or complications during hospitalization. Adjusted OR of 1 shows no difference from male patients. STEMI, ST-elevation myocardial infarction. MACCE, main adverse cardiovascular and cerebrovascular events.

As shown in Figure 2, female STEMI patients had doubled incidence of unadjusted in-hospital death (6.8% vs. 3.0%, *P* < 0.001), death or treatment withdrawal (14.5% vs. 5.6%, *P* < 0.001), and MACCE (18.5% vs. 9.4%, *P* < 0.001). Adjustment for clustering within hospitals in the generalized linear mixed model differ significantly between men and women with respect to in-hospital death (OR: 2.30, 95% CI: 1.71–3.11), death or treatment withdrawal (OR: 2.88, 95% CI: 2.32–3.57), and MACCE (OR: 2.27, 95% CI: 1.88–2.74) during hospitalization.

Table 3 shows the odds ratio of in-hospital outcomes in women over men with and without adjustment for a different set of variables, and the percent of the gender-associated risk difference accounted for by each set of explaining variables. The percent of the gender-associated risk difference accounted for by all study variables together was −53.08%, -63.83%, and 70.87% for in-hospital death, death or treatment withdrawal, and MACCE, and the residual odds ratios were statistically significant.

The variable that accounted for the most percent of the gender-associated risk difference was age (−58.46% for in-hospital death, −59.04% for death and treatment withdrawal, and −62.20% for MACCE) and cardiovascular risk factors (−40.77% for in-hospital death, −39.36% for death and treatment withdrawal, and -41.73% for MACCE); the results were consistent between the clinical outcomes.

Taken all the adjusted variables into the final model, gender disparities persist in death (OR: 1.61, 95% CI: 1.12–2.33), death or treatment withdrawal (OR: 1.68, 95% CI: 1.26–2.24), and MACCE (OR: 1.37, 95% CI: 1.08–1.74).

## 4. Discussion

In this registry study, we found marked sex differences in baseline characteristics, reperfusion therapy, treatment delay, and guideline-recommended acute drugs. Women experienced notably higher in-hospital death, death or treatment withdrawal, and MACCE, and the differences persisted after adjusted for covariates, old age, and cardiovascular risk factors, which explained the most sex-associated risk differences, were considered to be the major factor that influences in-hospital outcomes of female STEMI patients. In order to improve the prognosis of female STEMI patients, sex-based differences in patient characteristics and in-hospital outcomes stressed the importance of quality improvement efforts against female patients in clinical practice.

Prior studies showed female STEMI patients had higher in-hospital and external mortality, even among young patients, women with ACS had higher in-hospital mortality than their counterparts [7, 21–24]. Consistent with prior investigations, the sex differences among STEMI patients in the in-hospital outcomes were significant in our study, the rates of in-hospital clinical outcomes for women were nearly doubled, and what's more, the rates of adjusted in-hospital clinical outcomes were still higher in women. Female was an independent predictor of worse in-hospital clinical outcomes in STEMI patients independent of age, cardiovascular risk factors, and other covariates.

Confounder factors including old age and comorbidities were considered as reasons for sex differences [10, 12, 14, 15, 21–23]; our study observed that female STEMI patients were older and had a higher burden of cardiovascular risk factors like diabetes and hypertension. Age and cardiovascular risk factors play important roles in the sex gap among STEMI patients and were independent factors for worse clinical outcomes, which explained more than half of the disparity. The clinical characteristics of women notably differed from men. The vast majority of STEMI patients presented with typical chest pain symptoms; however, women were more likely to present with other additional symptoms such as frailty or back, shoulder, or neck pain [25], which may influence the time delays and diagnostic and management of myocardia infraction. Anterior location of STEMI increases the risk of death and postinfarction heart failure [26, 27]; women presented with anterior ST-elevation more commonly than their counterparts in our study. With an aging population and the rising burden of cardiovascular risk factors, persistent sex disparity in the STEMI must be of global concern; local and national efforts to advance primary and secondary prevention strategies may need to directly target women at high risk of heart attack.

Despite the international recommendations for equal treatment of women and men presenting with AMI [28], we found women were less likely to receive reperfusion therapy, and fibrinolysis was the main reperfusion strategy in female patients. Lower reperfusion rates in women have been reported since the early thrombolytic era, but this gap persists. We also observed that the proportion of successful reperfusion was significantly lower in women. Several reasons can be postulated to explain the issue, for instance, women with STEMI are more likely to have atypical symptoms possibly leading to delayed presentation and thus obstructing early reperfusion strategies at FMC, while reperfusion strategies are more efficient when they are initiated as early as possible [25], and women were more likely admitted to secondary hospital and fibrinolysis was the primary reperfusion technique among secondary hospital in China.

Longer time delays in the management of patients with STEMI were associated with in-hospital mortality [28, 29]. Previous work has shown that women have longer door-to-balloon times and longer door-to-needle times [30, 31]. We demonstrated that women are more likely to encounter delays from symptom onset to FMC, which has been previously found in other studies, and ultimately significantly delayed use of reperfusion therapies. Due to the successful construction of the dedicated regional networks such as chest pain centers, FMC-to-PCI delays were not observed in our study, while once the first medical contact has been made, the FMC-to-fibrinolysis time elapsed was still longer; considering the fibrinolysis was mainly taken in the secondary hospital, the reperfusion ability of secondary hospitals needs to be further improved.

Prior studies [10, 15, 21, 22, 32] indicated that both women and men derived similar reductions in morbidity and mortality with the prescriptions of aspirin, P2Y12 antagonist, and guidelines do not recommend differential use of these therapies based on sex [33, 34]. Our study found that the use of dual-antiplatelet therapy was still lower in women, which might have been caused by the fact that the combination of older age and severe comorbidities were more often present in females, thus possibly preventing the use of DAPT in clinical practice.

Our study indicates that, in order to eliminate the sex disparity among STEMI patients, more efforts should be made and better management of cardiovascular risk factors is vitally important. Efforts should also be made to ensure the evidence-based management of STEMI, such as PCI and DAPT, was applied in patients of both sexes. Women with STEMI in our study had prolonged prehospital delays, which likely contributed to lower rate of reperfusion and worse in-hospital outcomes. Health education targeting women to increase women's and their health providers' awareness and recognition of symptoms of STEMI should be implemented in pursuit of shortening the prehospital delay in women.

## 5. Limitations

Several limitations in our analysis should be considered. Firstly, the data collection burden for investigators may be the greatest barrier to the registry that may lead to some enrollment bias. We have carefully considered each element to limit the burden and have quality control measures in the registry. Secondly, our data are observational, the information mainly comes from medical records, and thus some degree of residual confounding cannot be excluded, and we only accounted for traditional clinical factors in our analysis. Thirdly, the geographical and organizational conditions of the Henan STEMI registry may not necessarily apply to other countries and regions.

## 6. Conclusion

Our study indicates that women hospitalized with STEMI had worse in-hospital outcomes, female STEMI patients were older and have a larger burden of cardiovascular risk factors, and women were less likely to be given reperfusion therapy and evidence-based acute drugs as compared with men. Old age and cardiovascular risk factors explained the most sex-related differences, and the sex disparity persists after being adjusted for confounder factors. This finding suggests that enhancing public awareness, better management of cardiovascular risk factors, and improved adherence to evidence-based management are still needed to close the gap.

## Figures and Tables

**Table 1:** Baseline characteristics of the study group: demographics, cardiovascular risk factors, medical history, and clinical characteristics at admission.

Variable	Men (*N* = 3829)	Women (*N* = 1234)	*P* value
Demographics			
Age (years)	60.8 (50.7, 68.7)	69.5 (62.3, 76.8)	<0.001
≥75 years	465 (12.1)	389 (31.5)	<0.001
Tertiary hospital	1969 (51.4)	541 (43.8)	<0.001
Married	3648 (95.3)	1065 (86.3)	<0.001
Farmer	2461 (64.3)	991 (80.3)	<0.001
Cardiovascular risk factors			
Hypertension	1575 (41.1)	658 (53.3)	<0.001
Dyslipidemia	2070 (54.1)	651 (52.8)	0.424
Diabetes	583 (15.2)	302 (24.5)	<0.001
Current smoker	1978 (51.7)	25 (2.0)	<0.001
Medical history			
Heart failure	31 (0.8)	23 (1.9)	0.002
Stroke	480 (12.5)	176 (14.3)	0.116
Coronary heart disease	253 (6.6)	61 (4.9)	0.035
CKD	20 (0.5)	12 (1.0)	0.083
Clinical characteristics			
Myocardial ischemia symptoms			<0.001
Typical	3261 (85.2)	992 (80.4)	
Atypical	553 (14.4)	239 (19.4)	
No symptom	15 (0.4)	3 (0.2)	
Cardiac arrest	125 (3.3)	29 (2.4)	0.104
Anterior myocardial infarction	2125 (55.5)	750 (60.8)	0.001
Killip class			<0.001
I	2853 (74.5)	791 (64.1)	
II	569 (14.9)	218 (17.7)	
III	204 (5.3)	112 (9.1)	
IV	203 (5.3)	113 (9.2)	
HR (beats per minute)			<0.001
<50	167 (4.4)	62 (5.0)	
50–109	3482 (90.9)	1073 (87.0)	
≥110	180 (4.7)	99 (8.0)	
SBP (mmHg)			0.044
<90	185 (4.8)	61 (4.9)	
90–139	2307 (60.3)	695 (56.3)	
≥140	1337 (34.9)	478 (38.7)	
cTnI*∗*	2.4 (0.4, 28.3)	4.1 (0.6, 38.0)	0.002
CK-MB*∗*	1.4 (0.63, 4.62)	1.8 (0.7, 5.2)	<0.001
Creatinine (*μ*mmol/L)	71 (62, 84)	59 (49, 74)	<0.001
LVEF (%)	56 (48, 61)	55 (46, 60)	0.008

Data are expressed as *n* (%) or median (IQR), unless otherwise noted. ^*∗*^The data are the multiple of the measured value and the upper limit of normal value. HR, heart rate; SBP, systolic blood pressure; cTnI, cardiac troponin I; CK-MB, creatine kinase-MB; and LVEF, left ventricular ejection fraction. Stroke contains ischemic and hemorrhagic. Coronary heart disease contains myocardial infarction, percutaneous coronary intervention, and coronary artery bypass graft.

**Table 2:** Treatment delay and use of guideline-recommended acute drugs between the study groups.

Variable	Men (*N* = 3829)	Women (*N* = 1234)	*P* value
Treatment delay			
Onset to FMC (minute)	190 (101, 540)	255 (120, 734)	<0.001
Onset to fibrinolysis (minute)^*∗*^	185 (125, 275)	218 (150, 339)	<0.001
FMC to fibrinolysis (minute)^*∗*^	45 (28, 78)	50 (31, 89)	0.047
Onset to PCI (minute)^*ϕ*^	243 (155, 390)	307 (188, 450)	<0.001
FMC to PCI (minute)^*ϕ*^	65 (42, 95)	62 (41, 95)	0.469
Guideline-recommended acute drugs			
Aspirin	3091 (80.7)	926 (75.0)	<0.001
P2Y12 antagonist	3214 (83.9)	965 (78.2)	<0.001
DAPT	3048 (79.6)	905 (73.3)	<0.001
Statin	639 (16.7)	246 (19.9)	0.009
Beta-blocker (24 h)	997 (26.0)	316 (25.6)	**0.764**

Data are expressed as *n* (%) and median (IQR) unless otherwise noted. ^*∗*^Onset to fibrinolysis and FMC to fibrinolysis were available among patients who were given fibrinolytic therapy. ^*ϕ*^Onset to PCI and FMC to PCI were available among patients who were received primary PCI. STEMI, ST-elevation myocardial infraction; FMC, first medical contact; PCI, percutaneous coronary intervention; P2Y12 antagonist, clopidogrel and ticagrelor; DAPT: dual-antiplatelet therapy; loading dose medicine: medicine used within 24 hours after being admitted.

**Table 3:** Odds ratio for in-hospital death, death or treatment withdrawal, and MACCE in women over men with adjustment for a different set of variables, and percent of the gender-associated hazard difference accounted by each set of explaining variables.

Variables adjusted for	In-hospital death			Death or treatment withdrawal			MACCE		
	Adjust OR (95% CI) (women vs. men)	*P* value	% of diff accounted	Adjust OR (95% CI) (women vs. men)	*P* value	% of diff accounted	Adjust OR (95% CI) (women vs. men)	*P* value	% of diff accounted
Base model	2.30 (1.71, 3.11)	<0.001	—	2.88 (2.32, 3.57)	<0.001	—	2.27 (1.88, 2.74)	<0.001	—
+Age	1.54 (1.13, 2.11)	0.007	−58.46	1.77 (1.41, 2.22)	<0.001	−59.04	1.48 (1.21, 1.81)	<0.001	−62.20
+Hospital grade	2.33 (1.73 3.13)	<0.001	2.31	2.86 (2.30, 3.56)	<0.001	−1.06	2.26 (1.87, 2.73)	<0.001	−0.79
+Socioeconomic status	2.41 (1.77, 3.28)	<0.001	8.46	2.71 (2.17, 3.39)	<0.001	−9.04	2.16 (1.78, 2.62)	<0.001	−8.66
+Cardiovascular risk factors	1.77 (1.27,2.46)	0.001	−40.77	2.14 (1.67, 2.73)	<0.001	−39.36	1.74 (1.40, 2.14)	<0.001	−41.73
+Medical history	2.26 (1.67, 3.05)	<0.001	−3.08	2.81 (2.26, 3.50)	<0.001	−3.72	2.25 (1.86, 2.72)	<0.001	−1.57
+ Clinical characteristics	2.19 (1.60, 2.99)	<0.001	−8.46	2.83 (2.24, 3.57)	<0.001	−2.66	2.19 (1.79, 2.68)	<0.001	−6.30
+Time to present	2.42 (1.80, 3.25)	<0.001	9.23	2.84 (2.28, 3.53)	<0.001	−2.13	2.25 (1.86, 2.72)	<0.001	−1.57
+Acute drugs	2.22 (1.65, 3.00)	<0.001	−6.15	2.74 (2.20, 3.41)	<0.001	−7.45	2.20 (1.82, 2.66)	<0.001	−5.51
+Reperfusion therapy	2.20 (1.62, 2.98)	<0.001	−7.69	2.70 (2.13, 3.35)	<0.001	−9.57	2.14 (1.76, 2.60)	<0.001	−10.24
All above	1.61 (1.12, 2.33)	0.012	−53.08	1.68 (1.26, 2.24)	<0.001	−63.83	1.37 (1.08, 1.74)	0.011	−70.87

Base model: adjusted for clustering of patients within hospitals. Age: continuous variables. Hospital grade: second hospital or tertiary hospital. Socioeconomic status: marriage status and occupation. Cardiovascular risk factors: hypertension, dyslipidemia, diabetes, and current smoker. Medical history: a medical history of coronary heart disease, heart failure, stroke, and chronic kidney diseases. Clinical characteristics: myocardial ischemia symptoms, heart rate, systolic blood pressure, Killip class, cardiac arrest, and anterior myocardial infarction at admission. Time to present: time from symptom onset to first medical contact at the hospital. Acute drugs: aspirin, P2Y12, and statin at emergency. Reperfusion therapy: successful reperfusion or not. Percent of sex difference account: (OR−unadjusted OR)/(unadjusted OR−1.0)^*∗*^100%.

## Data Availability

The identified participant data will not be shared. All data generated or analyzed during this study are included in this article.
